# Dihydromyricetin confers protection against myocardial ischemia-reperfusion injury by inhibiting ferroptosis through direct targeting of PPARα

**DOI:** 10.3389/fphar.2026.1794756

**Published:** 2026-04-10

**Authors:** Binwei Jin, Yang Zhang, Jiangbo Lin, Yu Zhu, Mengqi Yang, Yutao Zhang, Yanping Yin, Zhiming Zhang, Minjun Yang, Jianjun Jiang, Yafei Mi

**Affiliations:** 1 Department of Cardiology, Taizhou Hospital of Zhejiang Province affiliated to Wenzhou Medical University, Linhai, Zhejiang, China; 2 Laboratory of Cardiovascular Disease, Taizhou Hospital of Zhejiang Province affiliated to Wenzhou Medical University, Linhai, Zhejiang, China; 3 Medical Research Center, Taizhou Hospital of Zhejiang Province, Wenzhou Medical University, Linhai, Zhejiang, China; 4 Taizhou Hospital of Zhejiang Province, Shaoxing University, Linhai, China; 5 Department of Cardiology, Key Laboratory of Pan vascular Diseases of Wenzhou, The Second Affiliated Hospital and Yuying Children’s Hospital of Wenzhou Medical University, Wenzhou, China

**Keywords:** dihydromyricetin, ferroptosis, GPx4, myocardial ischemia-reperfusion injury, PPARα

## Abstract

**Background:**

Myocardial ischemia-reperfusion injury (MIRI) remains a therapeutic challenge with limited treatment options. Ferroptosis, characterized by lipid peroxidation, contributes significantly to MIRI pathogenesis. This study investigates whether dihydromyricetin (DHM), a bioactive flavonoid from *Ampelopsis grossedentata*, alleviates MIRI by inhibiting ferroptosis, and explores its cardioprotective mechanisms.

**Methods:**

A mouse myocardial I/R model was established *in vivo* by ligating the left anterior descending coronary artery for 30 min, followed by reperfusion for one or 7 days. Mice were pretreated with DHM (125 or 250 mg/kg, gavage) for 4 weeks, or with Fer-1 (10 mg/kg) for 1 week. *In vitro*, an OGD/R model was constructed using H9c2 cells, which were then treated with DHM (0–200 μM) or Fer-1 (10 μM). Potential targets were screened via network pharmacology. Target interaction was validated through biotin-pull-down, molecular docking, CETSA, and DARTS assays. Functional validation was performed using PPARα-knockdown and rescue cell lines.

**Results:**

GSEA indicated significant activation of the ferroptosis pathway in MIRI, and cellular experiments confirmed that OGD/R induced a typical ferroptosis phenotype. DHM dose-dependently reversed OGD/R-induced ferroptosis-related alterations and ameliorated mitochondrial ultrastructural damage. *In vivo*, DHM pretreatment significantly reduced serum CK-MB levels, myocardial infarct size, and histopathological injury in I/R mice, while reversing cardiac ferroptosis marker changes. Mechanistically, network pharmacology identified seven overlapping targets, among which DHM specifically reversed the downregulation of PPARα. Biochemical assays and molecular docking confirmed that DHM directly binds to and stabilizes PPARα via the E286 site. Knockdown of PPARα markedly attenuated the anti-ferroptotic and cardioprotective effects of DHM, whereas PPARα rescue partially restored its function.

**Conclusion:**

This study demonstrates for the first time that DHM alleviates ischemia-reperfusion injury by directly targeting the E286 site of PPARα, upregulating its levels, and thereby suppressing cardiomyocyte ferroptosis. This finding reveals a novel mechanism underlying the cardioprotective effect of DHM and provides a new theoretical basis for targeting the PPARα-ferroptosis axis in MIRI intervention.

## Introduction

Myocardial ischemia-reperfusion injury (MIRI) represents a major clinical challenge in the reperfusion therapy for acute myocardial infarction. Although timely restoration of blood flow is crucial for salvaging ischemic myocardium,the reperfusion process itself can induce additional myocardial damage, significantly impacting patient outcomes (Bax et al., 2019; Liu et al., 2024b). Reactive oxygen species (ROS) burst and mitochondrial dysfunction are recognized as central pathological events in MIRI (Xiang et al., 2024). The massive ROS generated during the initial moments of reperfusion attack cardiomyocytes, disrupt mitochondrial function, and ultimately lead to cell death. Despite various proposed intervention strategies, their clinical efficacy remains suboptimal, and specific therapeutic agents for preventing or treating MIRI are still lacking (Liu et al., 2021; Wang et al., 2022b; Cui et al., 2024; Yao et al., 2024; Chen et al., 2025b). Therefore, elucidating the molecular mechanisms of MIRI and developing novel therapeutic strategies hold significant clinical importance.

Ferroptosis is an iron-dependent form of regulated cell death characterized by intracellular iron overload, glutathione (GSH) depletion, and accumulation of lipid peroxides (Lillo-Moya et al., 2021; Jin et al., 2024). Morphologically, it presents with mitochondrial shrinkage, reduced cristae, and increased membrane density, while at the molecular level, it is closely associated with the inhibition of glutathione peroxidase 4 (GPX4) activity (Cui et al., 2024; Zhang et al., 2024). Recent studies have highlighted the critical role of ferroptosis in MIRI, involving iron-mediated ROS generation via the Fenton reaction, leading to peroxidation of polyunsaturated fatty acids (PUFAs) (Zhang et al., 2024). Accumulating evidence confirms that pharmacological inhibition of ferroptosis significantly attenuates MIRI-induced myocardial damage. For instance, fucoxanthin has been shown to exert cardioprotection via activation of the NRF2 signaling pathway, while isoliquiritigenin modulates the Nrf2/HO-1/SLC7A11/GPX4 axis (Yan et al., 2023; Yao et al., 2024), providing a mechanistic basis for targeting ferroptosis in MIRI treatment.

*Ampelopsis grossedentata*is a traditional medicinal plant, and its tender stems and leaves are processed into Tengcha, which is commonly used for anti-inflammatory treatments (Zhang et al., 2021). Modern pharmacological studies have shown that Tengcha is rich in flavonoids, with dihydromyricetin (DHM) being the most abundant component exhibiting a wide range of pharmacological activities (Zhang et al., 2018; Yan et al., 2024; Peng et al., 2025; Ye et al., 2025). Previous studies have reported the protective effects of DHM in various cardiac disease models, such as alleviating LPS-induced cardiomyocyte injury by inhibiting the TLR4/NF-κB pathway (Zhou et al., 2017), reducing oxidative stress and apoptosis in diabetic models (Wu et al., 2017), and counteracting doxorubicin-induced cardiotoxicity via the AMPK/mTOR pathway (Li X. et al., 2022). However, although preliminary studies suggest that DHM has protective potential against MIRI, its precise mechanisms of action remain incompletely understood (Liu et al., 2016; Wei et al., 2019; Li and Li, 2025).

Peroxisome proliferator-activated receptor alpha (PPARα) acts as a key regulator of cardiac energy metabolism, maintaining myocardial metabolic homeostasis by modulating the expression of fatty acid oxidation-related genes (Desvergne and Wahli, 1999). Studies indicate that PPARα expression is downregulated in cardiac hypertrophy and ischemia-reperfusion injury, accompanied by metabolic reprogramming (Barger et al., 2000). PPARα exhibits context-dependent effects: while excessive activation may promote ROS accumulation, its activation can also exert anti-inflammatory effects by suppressing the NF-κB pathway (Takano et al., 2000; Russell et al., 2005). Notably, PPARα crucially requires co-activation with PGC1α to regulate mitochondrial function, an axis essential for cardiac metabolic adaptation (Lehman et al., 2000; Rowe et al., 2010). Emerging multi-omics evidence suggests that PPARα activation alleviates MIRI by sustaining fatty acid β-oxidation (Gao et al., 2023), highlighting its therapeutic potential.

This study aimed to investigate the protective effect of DHM against MIRI and its underlying mechanism, with a focus on the ferroptosis pathway. The results demonstrated that DHM significantly alleviated MIRI in mice and improved ferroptosis-associated mitochondrial dysfunction. Mechanistically, DHM directly bound to PPARα and upregulated its levels, thereby inhibiting cardiomyocyte ferroptosis.

## Materials and methods

### Animal experiments

All animal experimental protocols were approved by the Animal Ethics Committee of Taizhou Enze Medical Center (Approval No: tzy-2025083) and conducted in accordance with EU Directive 2010/63/EU on animal welfare.

Sixty male C57BL/6 mice (6–8 weeks old, 15–20 g) were purchased from Shanghai SLAC Laboratory Animal Co., Ltd. (Shanghai, China). The animals were housed under controlled conditions (temperature: 23 °C ± 2 °C, humidity: 55% ± 5%, 12 h light/dark cycle) with free access to food and water, and acclimated for 1 week prior to experiments (Li et al., 2022a; Zhang et al., 2024b).

The ischemia-reperfusion (IR) model was established by ligating the left anterior descending coronary artery as previously described (Cai et al., 2023). Briefly, mice were anesthetized with 2% isoflurane, and the left anterior descending coronary artery was reversibly ligated for 30 min of ischemia followed by reperfusion. Assessments were performed at 24 h and 7 days after reperfusion.

The mice were divided into five groups: sham, IR, IR + low-dose DHM (125 mg/kg), IR + high-dose DHM (250 mg/kg), and IR + Fer-1 (10 mg/kg). DHM and Fer-1 were dissolved in a vehicle of 10% DMSO/90% corn oil. Fer-1 was administered intraperitoneally 7 days, 24 h, and 30 min before surgery (Zhang et al., 2025), while DHM was given daily by gavage for 4 weeks prior to surgery (Ye et al., 2025). The sham group received the vehicle only. All reagents were purchased from MedChemExpress (United States).

At the designated time points (24 h or 7 days post-reperfusion), mice were euthanized for tissue collection. Following anesthesia induced by inhalation of isoflurane (0.41 mL/min delivered under a fresh gas flow of 4 L/min in an induction chamber), euthanasia was performed via cervical dislocation in accordance with the United Kingdom Animal (Scientific Procedures) Act 1986. Heart tissues were subsequently harvested for further analysis.

### Cell culture and treatment

H9c2 (GNR5) cells and HEK-293T cells (GNHu43) were purchased from the Cell Bank of the Chinese Academy of Sciences (Shanghai, China). Rat primary cardiomyocytes (RCMCs) (RAT-iCell-c001) were purchased from the Cellverse Bioscience Technology Co., Ltd. (Shanghai, China). All cells were cultured in DMEM supplemented with 10% fetal bovine serum (Gibco, United States) and 1% penicillin–streptomycin at 37 °C under 5% CO_2_.

An oxygen–glucose deprivation/reoxygenation (OGD/R) model was utilized to simulate ischemia–reperfusion injury. Briefly, cells were washed with PBS and subjected to a 12-h pre-treatment with either DHM (25 μM) or Ferrostatin-1 (10 μM) in complete medium prior to OGD. Following pre-treatment, the medium was replaced with glucose-free HBSS buffer (containing 1.7 mM CaCl_2_, 3.5 mM KCl, 10 mM HEPES, 5 mM NaHCO_3_, 0.4 mM KH_2_PO_4_, 116 mM NaCl, and 0.8 mM MgSO_4_, pH 7.2–7.4). The cells were then transferred to a tri-gas incubator (95% N_2_, 5% CO_2_) and exposed to hypoxia for 3, 6, or 9 h; 9 h was selected for subsequent experiments based on preliminary data. After hypoxia, the buffer was removed, complete medium was replenished, and cells were returned to normoxic conditions for 18 h of reoxygenation.

The experiment consisted of two parts: (1) To investigate the protective effect of DHM on OGD/R-induced ferroptosis, wild-type H9c2 cells were divided into control, DHM-alone, OGD/R, OGD/R + DHM (co-treatment), and OGD/R + Fer-1 (co-treatment) groups; (2) To validate the functional necessity of PPARα and exclude potential off-target effects, rescue experiments were performed by transiently overexpressing PPARα in the knockdown cells. The groups included control, DHM, OGD/R, OGD/R + DHM (wild-type), OGD/R + DHM (knockdown), and OGD/R + DHM (knockdown + PPARα rescue) groups. DHM and Fer-1 were purchased from MedChemExpress (United States).

### CCK-8 assay

Cell viability was assessed using the CCK-8 assay. Briefly, cells were seeded in 96-well plates and cultured for 24 h before treatment. Subsequently, 10 μL of CCK-8 reagent (MedChemExpress, United States) was added to each well, followed by incubation at 37 °C for 1 h. The absorbance was measured at 450 nm using a microplate reader. Cell viability was normalized relative to the control group.

### Iron assay

Intracellular and myocardial Fe2+ levels were measured using an Iron Assay Kit (A039-2-1; Nanjing Jiancheng Bioengineering Institute, China) according to the manufacturer’s instructions.

### MDA and GSH assay

At the indicated time points, cells and murine myocardial tissue samples were collected. The contents of MDA and GSH were determined using commercial assay kits (MDA: A003-1; GSH: A006-2-1; Nanjing Jiancheng Bioengineering Institute, China) following the manufacturer’s protocols. The absorbance was measured at 532 nm and 405 nm, respectively, using a microplate reader.

### C11BODIPY fluorescence probe

Intracellular lipid ROS levels were detected using the C11-BODIPY fluorescent probe (D3861; Applied Biosystems, Thermo Fisher Scientific, United States). H9c2 cells were seeded in 48-well plates and incubated with 10 μM probe for 25 min in the dark. After three washes with PBS, images were captured using a fluorescence microscope.

### Western blotting

Western blot was performed as previously described (Zhang et al., 2024b). Total protein was extracted from H9c2 cells and heart tissues using RIPA lysis buffer (Beyotime, China) containing protease inhibitors (HY-K0011; MedChemExpress, United States). Protein concentration was determined by the BCA method (P0010S; Beyotime Institute of Biotechnology, Jiangsu, China). Equal amounts of protein were separated by electrophoresis (220 V, 30 min) using BeyoGel™ Plus PAGE (Tris-Gly, 10%, P0455S, Beyotime Biotechnology, Shanghai, China) with SWE rapid electrophoresis buffer (G2081, Servicebio, Wuhan, China), and transferred to PVDF membranes (0.45 μm; Millipore, United States) using rapid transfer buffer (Servicebio, G2028,Wuhan, China). After blocking with 5% non-fat milk for 1 h at room temperature, membranes were incubated overnight at 4 °C with the following primary antibodies:PPARα (1:1000; YM8234; ImmunoWay, United States),GPX4 (1:1000; HY-P80450; MedChemExpress, United States),SLC7A11 (1:1000; PAE410Ra01/PAE410Mu01; Cloud-Clone Corp, Wuhan, China),α-Tubulin (1:2000; #AF4651; Affinity Biosciences, Jiangsu, China),GAPDH (1:2000; AF1186, Beyotime Biotechnology, Shanghai, China),MAPK14 (1:1000; YM8276; ImmunoWay, United States),PPARγ (1:1000; YT3836; ImmunoWay, United States),SRC (1:1000; YM8471; ImmunoWay, United States),HIF1α (1:1000; YT2133; ImmunoWay, United States); ALOX12 (1:1000; HY-P82293; MedChemExpress, United States); TERT (1:1000; YT5514,ImmunoWay,United States); RIPK3(1:1000; TA7942; Abmart, Shanghai,China); Cleaved Caspase-3 (1:1000; T61532; Abmart, Shanghai, China).

Membranes were then incubated with HRP-conjugated secondary antibodies (1:4000, SA00001-1/SA00001-2,Proteintech/Wuhan Sanying, Wuhan, China) for 1 h at room temperature. Protein bands were visualized using ECL substrate (Super-Signal West Pico PLUS Chemiluminescent Substrate, Cat# 34580; Thermo Fisher Scientific, United States) and quantified with ImageJ software (National Institutes of Health, Bethesda, MD,United States).

### Real-time PCR assay

Total RNA was extracted from treated H9c2 cells using TRIzol reagent (Invitrogen,CA, United States) according to the manufacturer’s instructions. cDNA was synthesized from 1 μg of total RNA using the SuperScript First-Strand cDNA Synthesis Kit (Cat# 11904-018, Invitrogen,CA, United States). Quantitative real-time PCR was performed on the 7300 Plus Real-Time PCR System (Applied Biosystems, Thermo Fisher Scientific, United States) using SYBR Green Master Mix (Cat#A25742, Applied Biosystems, Thermo Fisher Scientific, United States) with gene-specific primers. Each reaction was performed in triplicate, and α-tubulin was used as the endogenous reference gene for normalization. Primer sequences are provided in Supplementary Table S1.

### Immunofluorescence staining

Cells were fixed with 4% paraformaldehyde for 15 min, washed with PBS, permeabilized with 0.5% Triton X-100 at room temperature for 10 min, and blocked with goat serum for 60 min. Subsequently, cells were incubated overnight at 4 °C with primary antibodies against SLC7A11 (1:200; #DF12509; Affinity Biosciences, Jiangsu, China) or GPX4 (1:200; #DF6701; Affinity Biosciences, Jiangsu, China). After PBS washing, cells were incubated with CoraLite488-conjugated goat anti-mouse IgG (SA00013-1,Proteintech/Wuhan Sanying, Wuhan, China) or CoraLite594-conjugated goat anti-rabbit IgG (SA00013-4,Proteintech/Wuhan Sanying, Wuhan, China) for 1 h at room temperature in the dark. Nuclei were stained with 0.1 μg/mL DAPI (D9564; Sigma-Aldrich, United States) for 5 min. Fluorescent images were captured using a Nikon Eclipse Ti2 microscope, and the fluorescence intensity of target proteins was quantified with ImageJ software.

### Transmission electron microscopy (TEM)

The mitochondrial ultrastructure of H9c2 cells was examined by transmission electron microscopy. Briefly, cells were double-fixed with 2.5% glutaraldehyde (4 °C overnight) and 1% osmium tetroxide (2 h), followed by dehydration through an ethanol gradient and embedding in Araldite resin. Ultrathin sections (60 nm) were double-stained with uranyl acetate and lead citrate, then observed and imaged under a transmission electron microscope.

### TTC/Evans Blue staining

Hearts were collected 24 h after reperfusion and perfused with 1 mL of 2% Evans Blue dye (E2129; Sigma-Aldrich, St. Louis, MO, United States) via the carotid artery to label the perfused myocardium. After washing away residual blood and dye, hearts were sectioned into 1 mm slices and incubated in 1% TTC solution (T109275; Aladdin Biochemical Technology Co., Ltd., Shanghai, China) at 37 °C for 15 min, followed by fixation with 4% paraformaldehyde for 24 h. Infarcted areas appeared white, risk zones stained red, and normal areas blue. The infarct size was analyzed using ImageJ software and expressed as a percentage of the infarct area relative to the area at risk.

### Detection of serum CK-MB

Blood samples were collected after ischemia-reperfusion, and serum was obtained by centrifugation (2000 × g, 20 min). The level of creatine kinase-MB isoenzyme was measured using a commercial ELISA kit (SEA479Mu; CLOUD-CLONE CORP, Wuhan, China) according to the manufacturer’s instructions.

### Heart tissue histological analysis

Mice were euthanized by overdose of isoflurane anesthesia. Heart tissues were collected, rinsed with PBS, and fixed in 4% paraformaldehyde. After paraffin embedding and sectioning (5 μm thickness), histological staining was performed using H&E, Masson’s trichrome, and WGA staining kits (Servicebio, Wuhan, China) according to the manufacturer’s instructions. Sections were observed and imaged under a fluorescence microscope (Leica, Germany) or light microscope. ImageJ software (National Institutes of Health, United States) was used for quantitative analysis of stained areas to evaluate cardiac morphology, fibrosis extent, and cardiomyocyte hypertrophy.

### Immunohistochemical analysis

Immunohistochemistry (IHC) was performed as previously described (Liu et al., 2022). Paraffin sections were deparaffinized in xylene, rehydrated through a graded ethanol series and PBS, then treated with 3% H_2_O_2_ and 0.1 M sodium citrate buffer (pH 6.0). After blocking with 10% goat serum at 37 °C for 1 h, sections were incubated with primary antibodies (SLC7A11 1:100, #DF12509, Affinity; GPX4 1:100, #DF6701, Affinity) overnight at 4 °C. Subsequently, sections were incubated with goat anti-rabbit secondary antibody at room temperature for 1 h, followed by PBS washing, DAB development (Absin, Shanghai, China), and hematoxylin counterstaining. Images were acquired using an Olympus CKX41 microscope.

### Pull-down assay

The experiment was conducted using the Pierce™ Biotinylated Protein Interaction Pull-Down Kit (Thermo Fisher). A total of 100 µL of biotinylated DHM (50 µg/100 µL) was incubated with 50 µL of streptavidin-agarose beads at 4 °C for 30 min, with biotin alone serving as the control. H9c2 cell lysate (>100 µL) was added and incubated at 4 °C with gentle shaking for 24 h. After centrifugation, the beads were washed three times. Prior to elution, 10 µL of neutralization buffer was added to each collection tube to adjust the sample pH. Then, 250 µL of elution buffer was added to each spin column, the cap was tightened, and the column was gently inverted 5–7 times. After standing at room temperature for 3–5 min, the column was centrifuged at 1250 *g* for 30–60 s. The eluate was collected, mixed with 5× loading buffer, boiled for denaturation, and analyzed by Western blot to detect interaction bands. Total lysate was used as the input control.

### Drug affinity responsive target stability (DARTS)

H9c2 cells were washed twice with PBS and lysed with RIPA buffer. The lysates were aliquoted and incubated with DMSO or different concentrations of DHM at room temperature for 1 h. Then, 20 mg/mL Pronase E (HY-114158A; MedChemExpress, United States) was added and incubated at room temperature for 20 min. The reaction was terminated by adding protein loading buffer, and the samples were prepared for subsequent analysis.

### Cellular thermal shift assay (CETSA)

293T cells were transfected with the plasmid pCDNA3.1-CMV-PPARα (p.E286A)-3xFLAG-hGHpolyA-EF1α-EGFP (OBiO Technology (Shanghai) Corp., Ltd., Shanghai, China) using Lipofectamine 3,000. After 48 h of transfection, with PPARα WT as the control, cells were treated with DHM or DMSO for 6 h. Cells were then collected in PBS containing 1% protease inhibitor and divided into eight groups. Samples were incubated at temperatures ranging from 37 °C to 72 °C (in 5 °C increments) for 3 min each, followed by two freeze-thaw cycles in liquid nitrogen. After centrifugation at 12,000 × g for 20 min, the supernatant was mixed with loading buffer, heated at 95 °C for 10 min, and subjected to Western blot analysis.

### Lentiviral transduction

Gene silencing was performed using a PPARα shRNA lentiviral vector (target sequence: “GCC​AAG​ATC​TGA​GAA​AGC​AAA”, Shanghai Bio-lifespan Co., LTD). H9c2 cells were seeded in 6-well plates and cultured for 24 h, followed by transduction with viral particles at a multiplicity of infection (MOI) of 20. After 48 h of incubation, cells were selected with puromycin (1 μg/mL) for 7 days, and PPARα knockdown efficiency was verified by Western blot.

### Small interfering RNA (siRNA) transfection

PPARα sense siRNA (5′-GAA​CAU​CGA​GUG​UCG​AAU​ATT-3′) and antisense siRNA (5′-UAU​UCG​ACA​CUC​GAU​GUU​CTT-3′) were purchased from OBiO Technology Corp., Ltd. (Shanghai, China). They were transfected into RCMCs cells with Lipofectamine 3,000 transfection reagent (Invitrogen, Carlsbad, CA, United States). After 4 h, the culture medium was replaced with DMEM supplemented with 10% fetal bovine serum (Gibco, United States).

### Plasmid transfection

The PPARα overexpression plasmid was obtained from OBiO Technology (Shanghai) Corp., Ltd. (Shanghai, China). The plasmid was transfected into PPARα-knockdown H9c2 or RCMCs cells using Lipofectamine 3,000 transfection reagent (Invitrogen, Carlsbad, CA, United States) following the manufacturer’s instructions. Twenty-four hours post-transfection, the medium was replaced with fresh high-glucose DMEM containing 10% fetal bovine serum (Gibco, United States).

### RNA-seq data and analysis

RNA-seq data were downloaded from the GEO database. Differentially expressed genes were identified using the R DEseq2 (version 1.40.2) (Anders and Huber, 2010) package. Gene expression data were further subjected to Gene Set Enrichment Analysis (GSEA) (Subramanian et al., 2005) GSEA software (version 4.3.2).

### Common targets of MIRI, ferroptosis, and DHM

To explore the role of DHM in treating MIRI and ferroptosis, a Venn diagram was constructed using R 4.2.3. The overlapping section represents potential therapeutic targets of DHM acting on both MIRI and ferroptosis.

### Molecular docking

The crystal structure of PPARα was derived from Protein Data Bank and the UniPort database. The 3D structure of Dihydromyricetin was downloaded from the PubChem database and optimized using the MMFF94 force field of OpenBabel software to obtain the optimal molecular structure with the lowest energy state. Before testing, the structures of PPARα and Dihydromyricetin were ensured to be in the optimized active arrangement. Finally, molecular docking was performed using Auto Dock Vina1.2.0.

### Data and statistical analysis

Statistical analysis was performed using GraphPad Prism 10.0 software. Data are presented as mean ± standard deviation. Intergroup comparisons were conducted by one-way analysis of variance (ANOVA) followed by Tukey’s post-hoc test. A P-value <0.05 was considered statistically significant, and significance levels are denoted as follows: *p < 0.05; **p < 0.01; ***p < 0.001; ****p < 0.0001; ns, no significance.

## Results

### The role of OGD/R treatment in inducing ferroptosis in H9C2 myocardial cells

**FIGURE 1:**
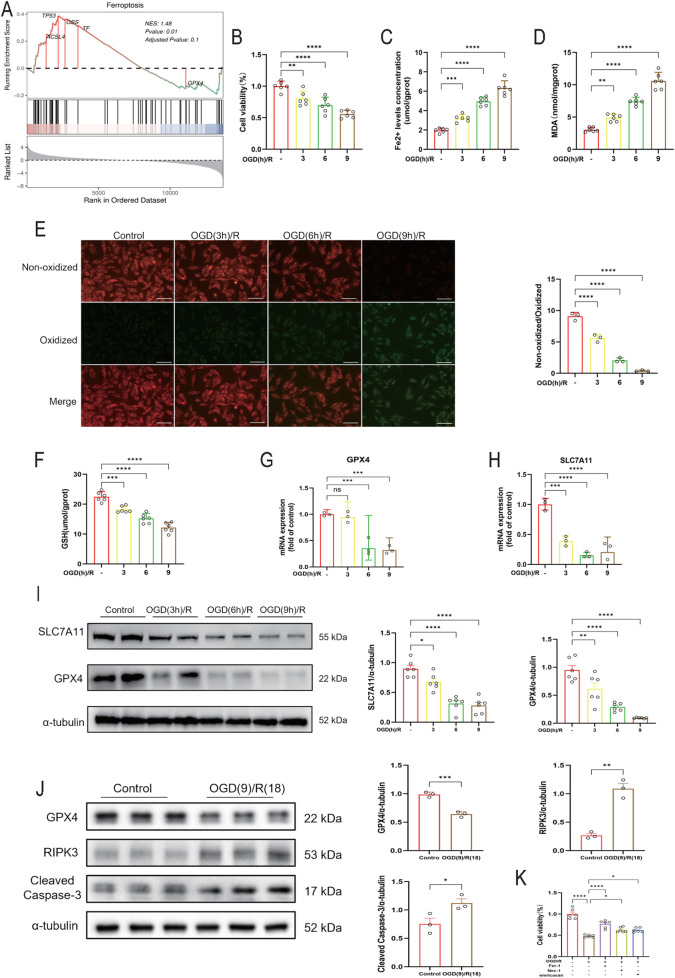
The role of OGD/R treatment in inducing ferroptosis in H9C2 myocardial cells. **(A)** Gene set enrichment analysis (GSEA) of ferroptosis-related genes in normal and MIRI rat hearts from the GSE240847 dataset. **(B)** CCK-8 assay was used to detect cell viability (n = 6 per group). **(C,D,F)** Fe2+ levels concentration, MDA and GSH levels in cells in different groups (n = 6 per group). **(E)** Representative fluorescent images and quantitative analysis of C11-BODIPY staining (n = 3 per group). Green and red fluorescence indicate oxidized and non-oxidized lipid, respectively. The green-to-red ratio was quantified in each group. Scale bar = 50 μm. **(G,H)** RT‒qPCR results of GPX4 and SLC7A11 in H9C2 cells subjected to OGD/R (n = 3 per group). **(I)** Western blot analysis was used to detect the expression of SLC7A11 and GPX4 proteins (n = 6 per group). **(J)** Western blot analysis was used to detect the levels of GPX4, RIPK3 and Cleaved Caspase-3 proteins (n = 3 per group). **(K)** CCK-8 assay was used to detect cell viability (n = 6 per group). The values were presented as mean ± SD. Statistical analysis was performed using one-way ANOVA. *p < 0.05; **p < 0.01; ***p < 0.001; ****p < 0.0001; ns, no significance.

To elucidate the pathogenesis of MIRI, we performed Gene Set Enrichment Analysis (GSEA) on gene expression profiles from normal and MIRI rat heart tissues in the public dataset GSE240847, using the human gene set WP_FERROPTOSIS. The results demonstrated a significant positive enrichment of the ferroptosis pathway during ischemia-reperfusion injury, indicating activation of this cell death process (Figure 1A). Subsequent validation in H9c2 cells revealed that OGD/R treatment induced a time-dependent decline in cell viability (Figure 1B) and a marked increase in intracellular Fe^2+^ levels (Figure 1C). Oxidative stress, a key pathophysiological factor in myocardial infarction, was significantly elevated, as evidenced by increased MDA content (Figure 1D). Furthermore, detection with the C11-BODIPY probe showed that OGD/R treatment substantially enhanced the production of lipid peroxides (Figure 1E). Compared to the control group, GSH levels were significantly reduced at all OGD/R time points (Figure 1F), further indicating redox imbalance. More importantly, prolonged OGD/R exposure led to a progressive decrease in both mRNA and protein expression levels of the key ferroptosis markers SLC7A11 and GPX4, suggesting suppression of their transcription and translation (Figures 1G–I).

To systematically investigate the cell death mechanisms in MIRI, we examined the activation of other programmed death pathways. Western blot analysis revealed that OGD/R treatment significantly upregulated the expression of the apoptosis marker Cleaved Caspase-3 and the necroptosis marker RIPK3 (Figure 1J), indicating concomitant activation of both apoptosis and necroptosis during MIRI. To compare the contribution of these distinct death pathways, specific inhibitors were applied. While the apoptosis inhibitor (emricasan) and necroptosis inhibitor (Nec-1) partially attenuated the OGD/R-induced loss of cell viability, the ferroptosis inhibitor (Ferrostatin-1) exerted the most pronounced protective effect (Figure 1K). These findings suggest that ferroptosis likely plays a pivotal role among the multiple programmed death modalities involved in MIRI, and targeting this pathway may represent an effective strategy for ameliorating myocardial ischemia-reperfusion injury.

### DHM suppresses OGD/R-induced ferroptosis in H9C2

**FIGURE 2:**
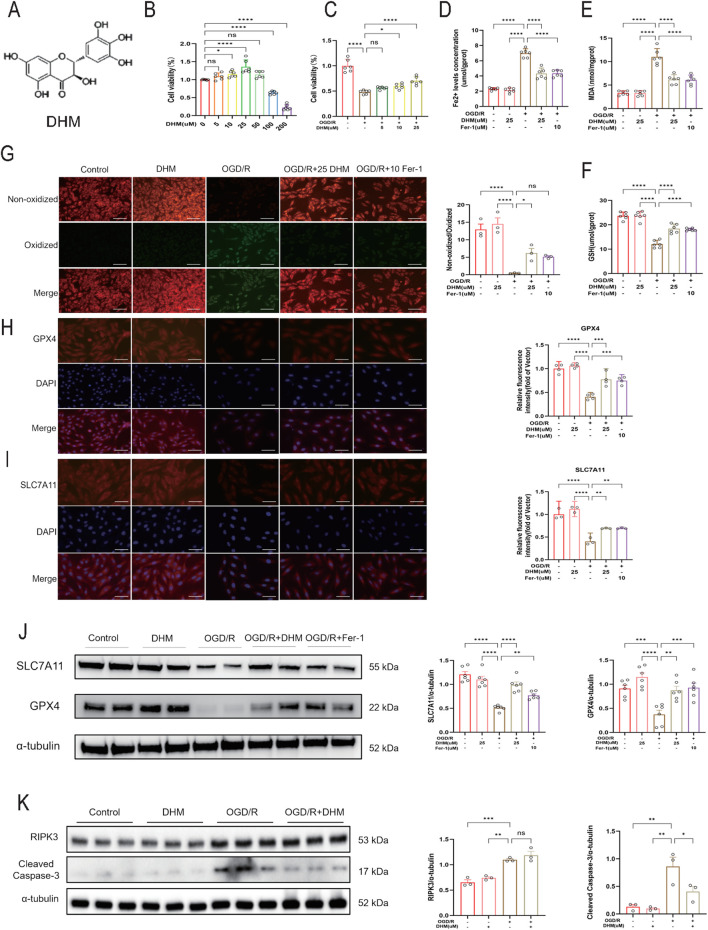
DHM suppresses OGD/R-induced ferroptosis in H9C2. **(A)** Chemical structure of DHM. **(B,C)** CCK-8 assay was used to detect cell viability (n = 6 per group). **(D–F)** Fe2+ levels concentration, MDA and GSH levels in cells in different groups (n = 6). **(G)** Representative fluorescent images and quantitative analysis of C11-BODIPY staining (n = 3 per group). Green and red fluorescence indicate oxidized and non-oxidized lipid, respectively. The green-to-red ratio was quantified in each group. Scale bar = 50 μm. **(H)** Representative images of GPX4 staining by IF in different groups (n = 4 per group) and Quantitative analysis of GPX4 levels. Scale Bar:50 μm. **(I)** Representative images of SLC7A11 staining by IF in different groups (n = 3 per group) and Quantitative analysis of SLC7A11 levels. Scale Bar:50 μm. **(J)** Western blot analysis was used to detect the levels of SLC7A11 and GPX4 proteins (n = 6 per group). **(K)** Western blot analysis was used to detect the levels of RIPK3 and Cleaved Caspase-3 proteins (n = 3 per group). The values were presented as mean ± SD. Statistical analysis was performed using one-way ANOVA. *p < 0.05; **p < 0.01; ***p < 0.001; ****p < 0.0001; ns, no significance.

DHM, a major bioactive flavonoid, exhibits multiple biological effects including antioxidant, anti-inflammatory, lipid- and glucose-regulating, and cardiovascular protective activities (Figure 2A). To investigate the role of DHM in regulating ferroptosis in cardiomyocytes, H9c2 cells subjected to OGD/R were treated with an optimal concentration of DHM, with the ferroptosis inhibitor Fer-1 used as a positive control. First, H9c2 cells were incubated with different concentrations of DHM (0, 5, 10, 20, 50, 100, and 200 μM). Cell viability was assessed using a CCK-8 kit, which showed that DHM at concentrations below 50 μM had no significant inhibitory effect on cell viability and even exhibited a slight promotive effect (Figure 2B). Compared with the OGD/R group, DHM treatment significantly enhanced H9c2 cell viability in a dose-dependent manner (Figure 2C) and markedly reduced intracellular Fe^2+^ levels (Figure 2D). In addition, MDA levels were significantly decreased, while GSH content was increased in the DHM + OGD/R group (Figures 2E,F). C11-BODIPY fluorescent probe detection revealed that DHM effectively suppressed lipid peroxide production (Figure 2G). Western blot and immunofluorescence analyses further confirmed that DHM treatment upregulated the levels of key ferroptosis-related proteins GPX4 and SLC7A11 (Figures 2H–J).

To further evaluate the potential modulation of non-ferroptotic pathways by DHM, Western blot analysis was performed to examine the expression of apoptosis- and necroptosis-associated proteins. The results showed that DHM significantly downregulated the protein level of Cleaved Caspase-3 in OGD/R-treated H9c2 cells, whereas no significant recovery effect on RIPK3 expression was observed (Figure 2K), suggesting that DHM may also exert protective effects against OGD/R injury via the apoptosis pathway. Thus, while inhibiting ferroptosis, DHM retains regulatory activity on non-ferroptotic pathways such as apoptosis, supporting that its protection arises from coordinated mitigation within a multimodal cell-death network rather than exclusive control of ferroptosis.

### DHM Modulates Mitochondrial Morphology in H9c2 cells subjected to OGD/R

**FIGURE 3:**
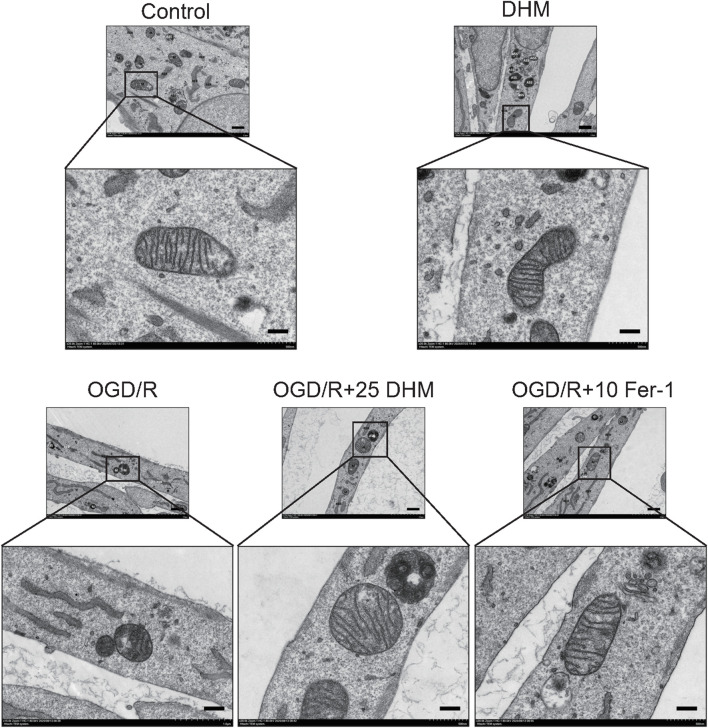
DHM Modulates Mitochondrial Morphology in H9c2 Cells Subjected to OGD/R. Transmission electron microscopy was used to observe the mitochondrial morphology associated with ferroptosis. M: Mitochondrion; ASS: Autophagolysosome; Go: Golgi apparatus; RER: Rough Endoplasmic Reticulum. (Scale bar = 2 μm, 1 µm).

Mitochondrial morphological changes were examined by transmission electron microscopy. As shown in Figure 3, mitochondria in the control and DHM-treated groups displayed intact oval or elongated morphology without significant rounding or shrinkage. The mitochondrial membranes remained continuous, and cristae were abundant and arranged in parallel arrays. In contrast, the OGD/R group exhibited severe ultrastructural damage, including mitochondrial rounding, reduction in size, increased membrane density, and extensive loss or fragmentation of cristae. Notably, these pathological alterations were markedly attenuated in the DHM + OGD/R group, with preserved mitochondrial shape, membrane integrity, and cristae structure. Similarly, the ferroptosis inhibitor Fer-1 also conferred a protective effect on mitochondrial ultrastructure in OGD/R-injured H9c2 cells.

### DHM-mediated inhibition of ferroptosis alleviates ischemia-reperfusion-induced cardiac injury

**FIGURE 4:**
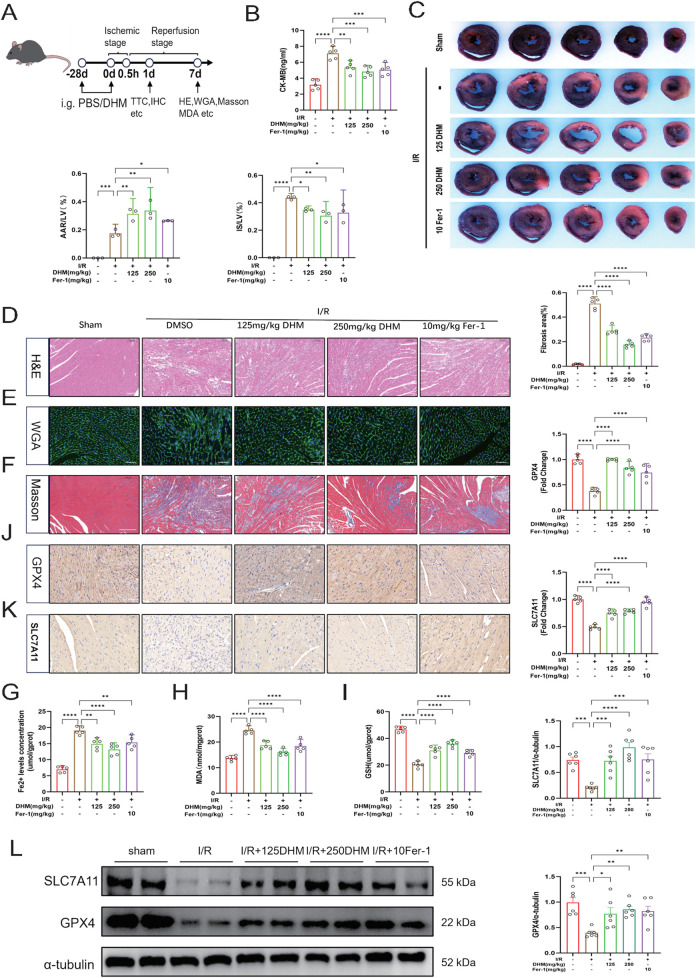
DHM-mediated inhibition of ferroptosis alleviates ischemia-reperfusion-induced cardiac injury. **(A)** Schematic diagram of the animal experimental process. The time points indicated in the image are representative. **(B)** Serum CK-MB levels (fivefold dilution) were determined in mice (n = 5 per group). **(C)** Representative ventricular cross-sections of hearts subjected to I/R, with or without DHM or Fer-1 treatment, stained with Evans Blue/TTC double staining. The red, blue, and white areas indicate the area at risk (TTC-stained), non-ischemic region (Evans Blue-stained), and infarct area, respectively. Quantitative analyses of IS/LV and AAR/LV were performed for each group (n = 3 per group). **(D)** Representative images of H&E staining (n = 5 per group, scale bar = 100 μm). **(E)** Representative images of WGA staining (n = 5 per group, scale bar = 50 μm). **(F)** Representative images and quantitative analysis of Masson’s trichrome staining (n = 5 per group, scale bar = 200 μm). **(G–I)** iron concentration, MDA and GSH levels in heart tissues (n = 5 per group). **(J,K)** IHC analysis of GPX4 and SLC7A11 levels in cardiac tissues from each group (representative images shown; scale bar = 50 μm; n = 5 per group), followed by quantitative assessment. **(L)** The protein levels of GPX4 and SLC7A11 in myocardial tissues were assessed by Western blotting (n = 6 per group), followed by quantitative analysis. The values were presented as mean ± SD. Statistical analysis was performed using one-way ANOVA. *p < 0.05; **p < 0.01; ***p < 0.001; ****p < 0.0001; ns, no significance.

To investigate the protective effect of DHM on MIRI, we established a murine myocardial I/R model and treated the animals with different doses of DHM (125 mg/kg and 250 mg/kg) alongside the ferroptosis inhibitor Fer-1 (10 mg/kg) as a positive control (Figure 4A). Compared with the I/R group, DHM treatment significantly reduced serum CK-MB activity, indicating partial reversal of myocardial injury (Figure 4B). Analysis of myocardial infarct size revealed that the I/R group had a significantly higher infarct proportion than the sham group, while DHM pretreatment markedly reduced the infarct area (Figure 4C). H&E staining revealed that I/R-induced myocardial structural damage, manifested as disordered myocardial fiber arrangement, reduced cardiomyocyte count, nuclear condensation, and inflammatory cell infiltration, was significantly ameliorated by DHM treatment (Figure 4D). WGA staining demonstrated a pronounced increase in cardiomyocyte area in the I/R group, which was suppressed by DHM administration (Figure 4E). Masson’s trichrome staining further confirmed that DHM effectively attenuated cardiac fibrosis induced by I/R (Figure 4F).

At the molecular level, DHM significantly inhibited the accumulation of Fe^2+^ and MDA and restored GSH levels in cardiac tissues of the I/R group (Figures 4G–I). Western blot and immunohistochemical analyses showed that I/R downregulated the levels of key ferroptosis-related proteins GPX4 and SLC7A11, an effect that was reversed by DHM pretreatment (Figures 4J–L). In conclusion, DHM alleviates I/R-induced myocardial injury by inhibiting ferroptosis, demonstrating potential cardioprotective effects.

### DHM directly targets and binds to PPARα, resulting in the upregulation of PPARα protein levels

**FIGURE 5:**
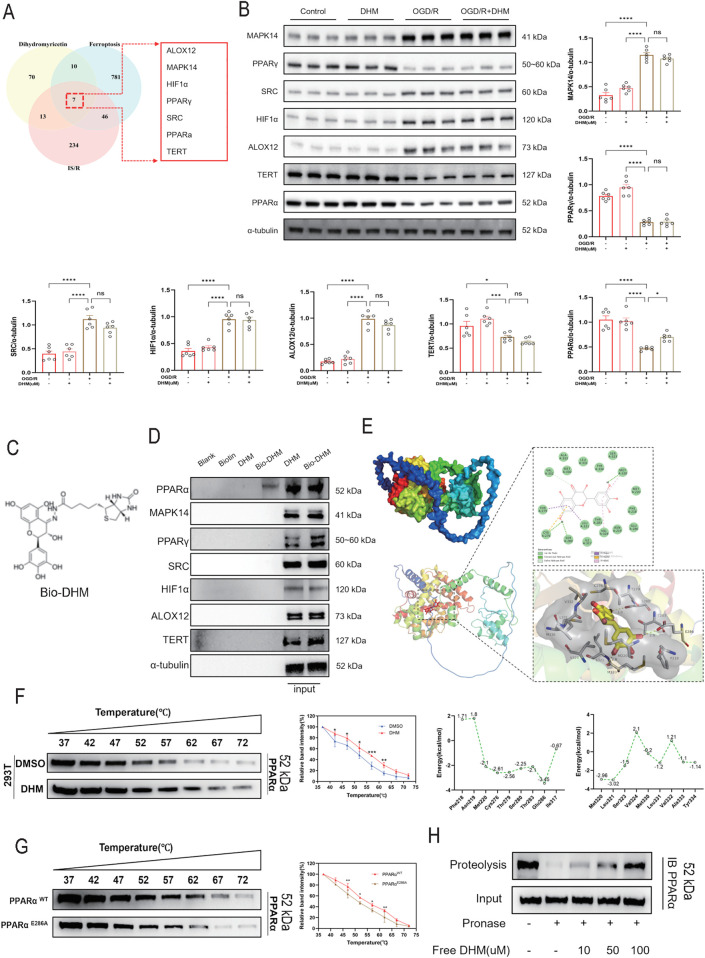
DHM directly targets and binds to PPARα, resulting in the upregulation of PPARα protein levels. **(A)** Venn diagram showing the overlap of potential DHM targets with MIRI-related and ferroptosis-related targets. **(B)** Western blot analysis was used to detect the levels of MAPK14,PPARγ,SRC,HIF1α,ALOX12,TERT and PPARα proteins (n = 6 per group). **(C)** Chemical structure of Bio-DHM. **(D)** A biotin-only condition served as the control. Bio-DHM was incubated with streptavidin-agarose beads, followed by addition of H9c2 cell lysates. After incubation, the eluate was collected for Western blot analysis, with total lysate used as the input control. **(E)** The binding pose of DHM to PPARα was determined by molecular docking. To further quantify the interaction, the per-residue energy contributions of key residues are presented in a line graph. **(F)** CETSA was performed to validate the binding of DHM to PPARα. 293T cells were treated with DHM or DMSO for 6 h. The cell lysates were aliquoted into eight samples, each heated at temperatures ranging from 37 °C to 72 °C (5 °C intervals) for 3 min, followed by two freeze-thaw cycles in liquid nitrogen. After centrifugation, the supernatants were subjected to Western blot analysis. The experiment was independently repeated three times. **(G)** Western blot analysis was used to assess the impact of DHM treatment on PPARαWT and PPARαE286A at different temperatures (n = 3). **(H)** Cell lysates were treated with DHM (0, 10, 50, and 100 μM) followed by Pronase E digestion. PPARα protein levels were then assessed by Western blot and quantified using ImageJ software. The values were presented as mean ± SD. Statistical analysis was performed using one-way ANOVA. *p < 0.05; **p < 0.01; ***p < 0.001; ****p < 0.0001; ns, no significance.

To investigate the molecular mechanism by which DHM inhibits I/R-induced cardiomyocyte ferroptosis, a network pharmacology approach was first employed. Potential targets of DHM (Supplementary Table S2), I/R-related genes, and ferroptosis-associated genes were subjected to intersection analysis, identifying seven candidate targets closely associated with both I/R injury and ferroptosis (Figure 5A). This analysis provided directional clues for subsequent mechanistic studies.

The impact of DHM intervention on the expression levels of these candidate targets was then assessed in OGD/R-treated H9c2 cells (Figure 5B). The results revealed a differential expression pattern induced by OGD/R treatment: the protein levels of MAPK14, SRC, HIF1α, and ALOX12 were significantly increased, while the levels of PPARγ, TERT, and PPARα was markedly decreased. Following DHM intervention, a specific and significant reversal of the OGD/R-induced downregulation was observed only for PPARα, restoring its level to near control levels. In contrast, DHM treatment did not significantly reverse the OGD/R-induced upregulation of MAPK14, SRC, HIF1α, and ALOX12, nor did it exhibit a pronounced restorative effect on the downregulated levels of PPARγ and TERT. These data indicate that, among the seven candidate targets, PPARα is a specifically regulated target of DHM in the context of I/R.

To validate the direct targeting of PPARα by DHM, a series of binding assays was performed. Biotin-labeled DHM specifically captured the PPARα protein from H9c2 cell lysates (Figures 5C,D). Molecular docking analysis demonstrated that DHM forms a stable binding complex with PPARα, with the E286 residue identified as crucial for maintaining this binding stability (Figure 5E). The CETSA further confirmed that DHM treatment significantly enhanced the thermal stability of PPARα (Figure 5F), an effect that was substantially attenuated by mutation of the E286 site (Figure 5G). Additionally, the DARTS assay showed that DHM protected PPARα from proteolysis by protease E (Figure 5H). Collectively, these results provide converging evidence that DHM specifically binds to PPARα via the E286 site. This direct interaction underlies the ability of DHM to specifically upregulate PPARα levels during I/R, thereby contributing to its protective effects against ischemia-reperfusion injury.

### PPARα is a key mediator in DHM-induced inhibition of ferroptosis

**FIGURE 6:**
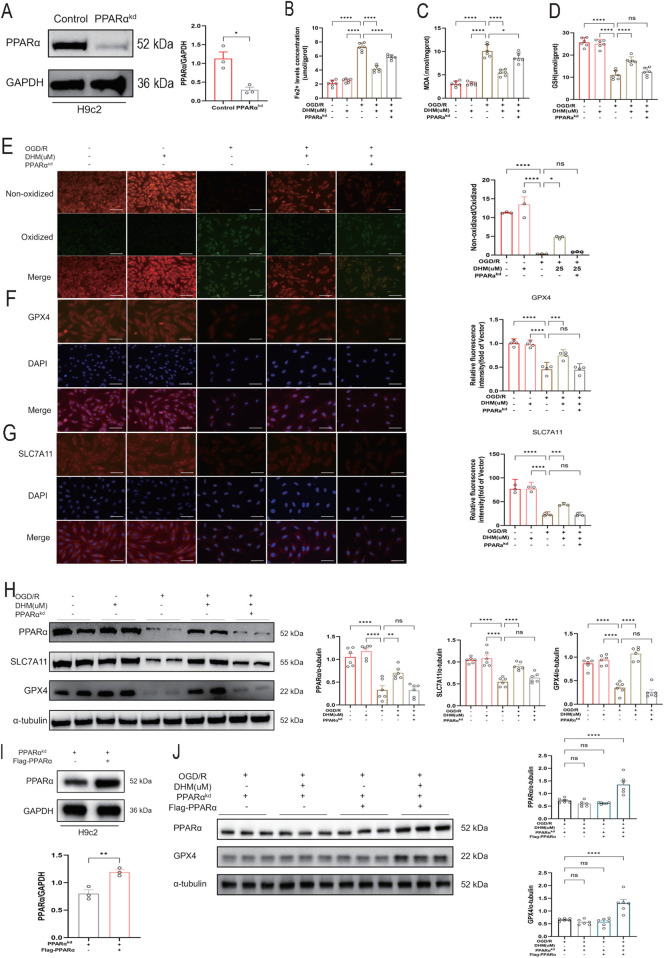
PPARα is a key mediator in DHM-induced inhibition of ferroptosis. **(A)** Western blot results of PPARα protein levels after PPARα shRNA transfection (n = 3 per group). **(B–D)** Fe2+ levels concentration, MDA and GSH levels in cells in different groups (n = 6 per group). **(E)** Representative fluorescent images and quantitative analysis of C11-BODIPY staining (n = 3 per group). Green and red fluorescence indicate oxidized and non-oxidized lipid, respectively. The green-to-red ratio was quantified in each group. Scale bar = 50 μm. **(F)** Representative images of GPX4 staining by IF in different groups (n = 4 per group) and Quantitative analysis of GPX4 levels. Scale Bar:50 μm. **(G)** Representative images of SLC7A11 staining by IF in different groups (n = 3 per group) and Quantitative analysis of SLC7A11 levels. Scale Bar:50 μm. **(H)** Western blot analysis was used to detect the levels of SLC7A11, GPX4 and PPARα proteins (n = 6 per group). **(I)** Western blot analysis of PPARα protein levels in PPARα-knockdown (KD) H9c2 cells transfected with a PPARα-overexpressing plasmid. (n = 3 per group). **(J)** Western blot analysis was used to detect the levels of GPX4 and PPARαproteins (n = 6 per group). The values were presented as mean ± SD. Statistical analysis was performed using one-way ANOVA. *p < 0.05; **p < 0.01; ***p < 0.001; ****p < 0.0001; ns, no significance.

To define the functional role of PPARα in the anti-ferroptotic action of DHM, a stable PPARα-knockdown H9c2 cell line (PPARα^KD^) was established, with its knockdown efficiency confirmed by Western blot analysis (Figure 6A). To address the potential for off-target effects inherent in single-shRNA knockdown models, a rescue experiment was performed by overexpressing PPARα via plasmid transfection in the PPARα^KD^ cells, successfully restoring PPARα levels (Figure 6I).

OGD/R treatment significantly increased intracellular levels of Fe^2+^ and MDA while decreasing GSH content. DHM intervention effectively reversed these alterations. However, in PPARα^KD^ cells, the ameliorative effects of DHM on these ferroptosis-related biochemical markers were markedly attenuated (Figures 6B–D). C11-BODIPY fluorescence staining further demonstrated that DHM significantly inhibited OGD/R-induced lipid peroxidation, but this inhibitory effect was abolished in PPARα^KD^ cells (Figure 6E).

To further investigate the dependence of DHM’s protective effect against ferroptosis on PPARα, the levels of core ferroptosis-related proteins, GPX4 and SLC7A11, was examined. The upregulation of GPX4 and SLC7A11 levels by DHM was significantly inhibited in PPARα^KD^ cells (Figures 6F–H). Importantly, in the rescue cells (PPARα^KD+OE^), DHM restored its ability to upregulate GPX4 levels (Figure 6J). These results indicate that the loss of PPARα impairs the protective effect of DHM against ferroptosis, while re-expression of PPARα can partially restore this effect. This genetic evidence establishes PPARα as an indispensable target protein for DHM in inhibiting ferroptosis.

### Validation of PPARα necessity for DHM-mediated anti-ferroptotic Effects in Rat Primary Cardiomyocytes (RCMCs)

**FIGURE 7:**
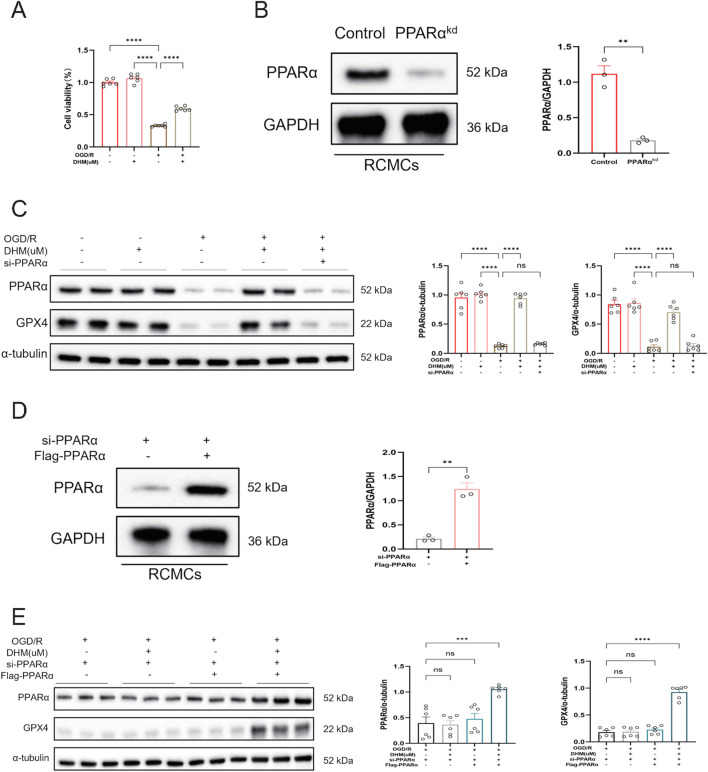
Validation of PPARα Necessity for DHM-mediated Anti-ferroptotic Effects in Rat Primary Cardiomyocytes (RCMCs). **(A)** CCK-8 assay was used to detect cell viability (n = 6 per group). **(B)** Western blot analysis was used to detect the levels of PPARα proteins (n = 3 per group). **(C)** Western blot analysis was used to detect the levels of GPX4 and PPARα proteins (n = 6 per group). **(D)** Western blot analysis was used to detect the levels of PPARα proteins (n = 3 per group). **(E)** Western blot analysis was used to detect the levels of GPX4 and PPARα proteins (n = 6 per group). The values were presented as mean ± SD. Statistical analysis was performed using one-way ANOVA. *p < 0.05; **p < 0.01; ***p < 0.001; ****p < 0.0001; ns, no significance.

To further validate the role of PPARα in DHM-mediated ferroptosis inhibition, supplementary experiments were performed in RCMCs. Cell viability assays demonstrated that DHM intervention significantly alleviated OGD/R-induced injury in RCMCs (Figure 7A). To determine the dependence of this protective effect on PPARα, a PPARα-knockdown RCMC model was established (Figure 7B), and changes in the expression of the core ferroptosis protein GPX4 were examined. The results showed that DHM significantly reversed the OGD/R-induced downregulation of GPX4 protein levels, but this restorative effect was abolished in PPARα-knockdown cells (Figure 7C). To exclude potential off-target effects of single siRNA knockdown, PPARα was re-expressed in the knockdown cells through rescue experiments (Figure 7D). The results indicated that DHM regained its ability to upregulate GPX4 protein levels after PPARα rescue (Figure 7E). These findings further confirm in primary cardiomyocytes that PPARα is an indispensable target for DHM to exert its anti-ferroptotic effect.

## Discussion

**FIGURE 8:**
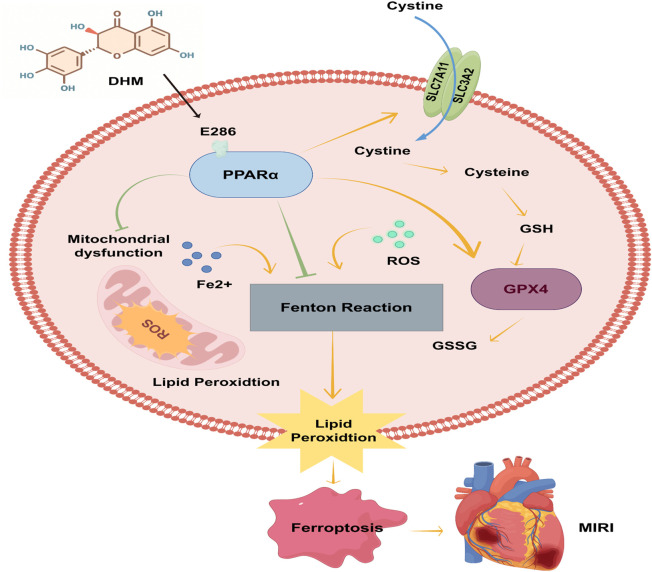
DHM alleviates MIRI by specifically binding to the E286 site of PPARα and upregulating its levels, thereby suppressing excessive ROS generation and Fe^2+^ accumulation, blocking lipid peroxidation and ferroptosis, and attenuating mitochondrial damage.

The clinical management of MIRI remains a major challenge in cardiology, with no consensus on optimal therapeutic strategies (Welt et al., 2024; Zhang et al., 2024a). Current interventions focus on ischemic preconditioning, pharmacological approaches (e.g., antioxidants, calcium channel blockers), and microcirculation protection post-reperfusion (Liu et al., 2018; Chen et al., 2023; Liu et al., 2024a; Welt et al., 2024). However, due to the complex pathophysiology of MIRI involving oxidative burst, calcium overload, and inflammatory responses, existing methods are limited by single-target actions, narrow therapeutic windows (e.g., the critical ROS burst phase during early reperfusion), and potential systemic side effects (e.g., hypotension induced by calcium channel blockers) (Jin et al., 2024; Chen et al., 2025a). Thus, developing novel interventions with high efficacy and low toxicity is of significant clinical value. This study demonstrates that DHM pretreatment significantly suppresses excessive ROS generation and Fe^2+^ accumulation, inhibits lipid peroxidation and ferroptosis, thereby alleviating MIRI-induced myocardial injury. Notably, we identify for the first time that DHM exerts cardioprotection by specifically binding to the E286 site of PPARα and upregulating its protein expression. These findings are illustrated in Figure 8.

Emerging evidence has confirmed the critical role of ferroptosis in MIRI (Li et al., 2021; Ma et al., 2022; Wang et al., 2022a; Han et al., 2023). With ongoing research, the regulatory network of ferroptosis in MIRI is being systematically elucidated, revealing the involvement of key transcription factors and signaling pathways. Through systematic experimental validation, Li et al. (2025) demonstrated that the expression of transcription factor ETS1 was significantly upregulated in a MIRI model, promoting ferroptosis by specifically regulating its downstream effector PIM3. Genetic inhibition of ETS1 notably increased the expression of key antioxidant proteins such as GPX4 and SLC7A11, thereby effectively mitigating myocardial injury (Li et al., 2025). Notably, the team of Professors Zhang et al. (2025) recently elucidated a novel “autophagy-dependent ferroptosis” molecular pathway: ischemia-reperfusion leads to abnormal cytoplasmic double-stranded DNA accumulation, activating the cGAS-STING innate immune pathway. Mechanistically, activated STING protein directly binds to GPX4 and promotes its degradation via autophagy, ultimately inducing ferroptosis (Wang et al., 2025). This discovery not only establishes a molecular link among innate immunity, autophagy, and ferroptosis for the first time but also provides new insights into the complex pathological mechanisms of MIRI.

This study integrated network pharmacology analysis, bioinformatics, and *in vivo*/*in vitro* experiments to systematically investigate the effect of DHM on ferroptosis in MIRI.

Consistent with previous studies, the ferroptosis inhibitor Fer-1 significantly ameliorated OGD/R-induced injury in H9c2 cardiomyocytes, as evidenced by restored cell viability, reduced Fe^2+^ accumulation, decreased lipid peroxidation, preserved mitochondrial ultrastructure, and upregulated levels of key ferroptosis regulatory proteins GPX4 and SLC7A11, further supporting ferroptosis inhibition as a potential therapeutic strategy against MIRI. This study confirms the substantial protective effect of DHM on MIRI. In animal experiments, DHM pretreatment reduced serum CK-MB activity, infarct size, histopathological damage, myocardial hypertrophy, and cardiac fibrosis in I/R mice. In cellular assays, DHM dose-dependently increased viability, decreased Fe^2+^ accumulation and lipid peroxidation, and maintained mitochondrial integrity in OGD/R-treated H9c2 cells. Previous research indicates that DHM exerts cardioprotective effects by reducing infarct size, improving cardiac function, and preserving mitochondrial integrity (Wei et al., 2019), and reduces arrhythmia susceptibility in coronary ligation models (Liu et al., 2016). Mechanistically, this study demonstrates that DHM directly targets and binds to PPARα, upregulating the levels of GPX4 and SLC7A11. In PPARα-knockdown H9c2 cells, the inhibitory effect of DHM on OGD/R-induced lipid peroxidation and its ability to upregulate GPX4 levels were both significantly attenuated. Furthermore, a rescue experiment demonstrated that re-expressing PPARα in the knockdown cells partially restored DHM’s capacity to upregulate GPX4 levels. Notably, this finding was further validated in rat primary cardiomyocytes. These results confirm that PPARα is a specific and key mediator through which DHM exerts its anti-ferroptotic effect. While prior studies suggest PPARα activation protects against MIRI (Gao et al., 2023), this study provides genetic evidence that PPARα is a key direct target and mediator in DHM-mediated inhibition of ferroptosis.

This study is the first to systematically elucidate a novel molecular target and precise mechanism underlying the cardioprotective effect of DHM against MIRI. Although DHM is known to confer multi-faceted cardiovascular benefits, its direct protein target and the exact molecular mode of binding remained unclear. Our work began by integrating multiple databases to identify seven candidate targets closely associated with both MIRI and ferroptosis. Subsequent experimental validation moved beyond correlative analysis, as a biotin-labeled pull-down assay directly confirmed a physical interaction between DHM and PPARα, laying a solid foundation for mechanistic investigation.

Building on this, the core novelty of our work lies not only in identifying PPARα as a direct target of DHM, but also in precisely defining the key binding residue—E286—through molecular docking simulations. Binding free energy calculations and interaction analysis revealed that the specific scaffold of DHM forms a stable network of hydrogen bonds and hydrophobic interactions with the E286 site within the PPARα ligand-binding domain, a binding mode distinct among reported natural products. To functionally validate this prediction, orthogonal assays—CETSA and DARTS—were employed. The results demonstrated that DHM treatment significantly enhanced the thermal stability and protease resistance of PPARα, an effect that was markedly attenuated by mutation of the E286 site. This experimentally confirms that E286 is the key specific site through which DHM acts as a molecular stabilizer, locking and activating the PPARα protein conformation.

Collectively, this work goes beyond a conventional compound-target association. It reveals, at atomic resolution, the precise structural basis of the DHM-PPARα interaction and establishes a complete evidence chain from *in silico*simulation to cellular functional validation. This finding not only provides unprecedented molecular detail for DHM’s cardioprotective mechanism, but also offers a new theoretical basis and a potential direction for future structure-guided drug design or the screening of high-affinity ligands targeting PPARα, specifically via the E286 site.

The activation status of PPARα is closely associated with cardiomyocyte susceptibility to ferroptosis, suggesting its potential role as a molecular bridge connecting metabolic regulation and cell death pathways (Zhao et al., 2025). Recent studies indicate that PPARα can indirectly suppress ferroptosis by maintaining mitochondrial function. For example, Zhu et al. (2023) demonstrated in a sepsis-induced cardiac dysfunction model that cardiomyocyte-specific (but not myeloid-specific) PPARα activation improved mitochondrial dysfunction (e.g., enhanced ATP production, restored mitochondrial complex activity), reduced ROS generation, and suppressed IL-6/STAT3/NF-κB-mediated inflammation, thereby ameliorating cardiac dysfunction (Zhu et al., 2023). Another study confirmed that the PPARα agonist fenofibrate enhances antioxidant defense via the PPARα/Nrf2 pathway while restoring mitophagy, ultimately exerting cardioprotective effects (Hu et al., 2025). In this study, we established a stable PPARα-knockdown H9c2 cell model to evaluate whether DHM’s inhibition of MIRI-induced ferroptosis depends on PPARα. Results showed that PPARα knockdown significantly attenuated DHM’s suppressive effects on ferroptosis, further confirming PPARα′s critical role in mediating DHM’s anti-ferroptotic pathway.

Although this study provides valuable insights, several limitations should be acknowledged: (1) While the cardioprotective effect of DHM has been demonstrated in rodent models and cellular experiments, its clinical translatability requires further validation in human-derived cellular models, animal models with comorbidities, and large-animal studies; (2) Whether the binding of DHM to the E286 site of PPARα modulates its post-translational modifications, such as phosphorylation or ubiquitination, remains unclear and warrants further investigation; (3) The involvement of downstream mediator proteins of PPARα in the ferroptosis pathway needs to be further elucidated; (4) Systematic studies are needed to explore whether DHM exerts cardioprotective effects via other potential targets beyond PPARα; (5) Although DHM significantly improved cardiac biomarkers, infarct size, and tissue structure in the I/R injury model, the present study lacks functional imaging (e.g., echocardiography) or hemodynamic data (e.g., LVEF, dP/dt), thus lacking direct evidence for ventricular functional recovery. Therefore, whether DHM indeed translates into clinically meaningful overall cardiac functional improvement requires future studies incorporating relevant functional assessments; (6) Only male mice were used to minimize hormonal variability, particularly from estrogen fluctuations. This limits the generalizability of the findings, as sex is a key biological variable in cardiovascular studies. Future work should include both sexes to fully assess the translational potential of DHM.

## Conclusion

This study demonstrates for the first time that DHM attenuates ischemia-reperfusion injury by directly targeting and binding to the E286 site of PPARα, upregulating its levels, and thereby suppressing myocardial ferroptosis. This finding provides molecular-level experimental evidence for the cardioprotective effect of DHM and represents a preclinical proof of concept. It should be noted that the dosage and administration regimen employed in this study are exploratory designs based on animal and cellular models, and the pharmacokinetic profile, safety, and effective therapeutic window in humans remain unclear. Therefore, before advancing further translational research, it is necessary to systematically evaluate the pharmacokinetics, toxicology, and dose-response relationships of DHM in large animal models or early-phase clinical trials to provide a reliable basis for its subsequent development.

## Data Availability

The original contributions presented in the study are included in the article/Supplementary Material, further inquiries can be directed to the corresponding authors.
