# Supplementary material for: Dihydromyricetin confers protection against myocardial ischemia-reperfusion injury by inhibiting ferroptosis through direct targeting of PPARα
Source: Front Pharmacol. 2026 Apr 10;17:1794756. doi: 10.3389/fphar.2026.1794756 (PMC13105976; doi:10.3389/fphar.2026.1794756)
Supplement: Supplementary file 1 [file Table1.docx]

**Supplementary Material**

Table S1: Primers for RT-PCR.

| **Gene** | **Forward primer** | **Reverse primer** |
| --- | --- | --- |
| α-tubulin | agatttgatggggccctgaa | caggcattggtgatctctgc |
| GPX4 | cgatctgcatgcccgatatg | ggcatcgtccccatttacac |
| SLC7A11 | gtctgcctgtggagtactgt | attacgagcagttccaccca |
