# Supplementary material for: Dihydromyricetin confers protection against myocardial ischemia-reperfusion injury by inhibiting ferroptosis through direct targeting of PPARα
Source: Front Pharmacol. 2026 Apr 10;17:1794756. doi: 10.3389/fphar.2026.1794756 (PMC13105976; doi:10.3389/fphar.2026.1794756)
Supplement: Supplementary file 2 [file Table2.docx]

**Supplementary Material**

Tabel S2 SwissTarget Prediction

| Target | Common name | Uniprot ID |
| --- | --- | --- |
| Matrix metalloproteinase 13 | MMP13 | P45452 |
| Matrix metalloproteinase 12 | MMP12 | P39900 |
| Carbonic anhydrase VII | CA7 | P43166 |
| Carbonic anhydrase XII | CA12 | O43570 |
| Carbonic anhydrase IV | CA4 | P22748 |
| Cytochrome P450 1B1 | CYP1B1 | Q16678 |
| Carbonic anhydrase XIII | CA13 | Q8N1Q1 |
| P-glycoprotein 1 (by homology) | ABCB1 | P08183 |
| Multidrug resistance-associated protein 1 | ABCC1 | P33527 |
| Estradiol 17-beta-dehydrogenase 1 | HSD17B1 | P14061 |
| Testis-specific androgen-binding protein | SHBG | P04278 |
| Carbonyl reductase [NADPH] 1 | CBR1 | P16152 |
| Cyclooxygenase-1 | PTGS1 | P23219 |
| Beta-secretase 1 | BACE1 | P56817 |
| Estrogen receptor alpha | ESR1 | P03372 |
| Kallikrein 1 | KLK1 | P06870 |
| Kallikrein 2 | KLK2 | P20151 |
| Stem cell growth factor receptor | KIT | P10721 |
| Tyrosine-protein kinase SRC | SRC | P12931 |
| Vascular endothelial growth factor receptor 2 | KDR | P35968 |
| Fibroblast growth factor receptor 1 | FGFR1 | P11362 |
| Hepatocyte growth factor receptor | MET | P08581 |
| Carbonic anhydrase II | CA2 | P00918 |
| Carbonic anhydrase I | CA1 | P00915 |
| Carbonic anhydrase III | CA3 | P07451 |
| Carbonic anhydrase VI | CA6 | P23280 |
| Carbonic anhydrase VB | CA5B | Q9Y2D0 |
| Carbonic anhydrase VA | CA5A | P35218 |
| Taste receptor type 2 member 31 | TAS2R31 | P59538 |
| ATP-binding cassette sub-family G member 2 | ABCG2 | Q9UNQ0 |
| Monoamine oxidase B | MAOB | P27338 |
| Carbonic anhydrase IX | CA9 | Q16790 |
| Microtubule-associated protein tau | MAPT | P10636 |
| DNA (cytosine-5)-methyltransferase 1 | DNMT1 | P26358 |
| Dual-specificity tyrosine-phosphorylation regulated kinase 1A | DYRK1A | Q13627 |
| HERG | KCNH2 | Q12809 |
| Beta amyloid A4 protein | APP | P05067 |
| MAP kinase p38 alpha | MAPK14 | Q16539 |
| Telomerase reverse transcriptase | TERT | O14746 |
| Matrix metalloproteinase 2 | MMP2 | P08253 |
| 6-phosphogluconate dehydrogenase | PGD | P52209 |
| CMP-N-acetylneuraminate-beta-1,4-galactoside alpha-2,3-sialyltransferase | ST3GAL3 | Q11203 |
| Alpha-(1,3)-fucosyltransferase 7 | FUT7 | Q11130 |
| Matrix metalloproteinase 14 | MMP14 | P50281 |
| Fucosyltransferase 4 | FUT4 | P22083 |
| Signal transducer and activator of transcription 1-alpha/beta | STAT1 | P42224 |
| Squalene monooxygenase (by homology) | SQLE | Q14534 |
| Retinoid X receptor alpha | RXRA | P19793 |
| Adenosine A1 receptor (by homology) | ADORA1 | P30542 |
| Cytochrome P450 19A1 | CYP19A1 | P11511 |
| Acetylcholinesterase | ACHE | P22303 |
| Phospholipase A2 group 1B | PLA2G1B | P04054 |
| Hypoxia-inducible factor 1 alpha | HIF1A | Q16665 |
| Acyl coenzyme A:cholesterol acyltransferase | CES1 | P23141 |
| Peroxisome proliferator-activated receptor gamma | PPARG | P37231 |
| Carboxylesterase 2 | CES2 | O00748 |
| Placenta growth factor | PGF | P49763 |
| Vascular endothelial growth factor A | VEGFA | P15692 |
| Adenosine A3 receptor | ADORA3 | P0DMS8 |
| Apoptosis regulator Bcl-2 | BCL2 | P10415 |
| Metabotropic glutamate receptor 5 | GRM5 | P41594 |
| DNA polymerase beta (by homology) | POLB | P06746 |
| Phospholipase A2 group IIA | PLA2G2A | P14555 |
| Phospholipase A2 group V | PLA2G5 | P39877 |
| Group X secretory phospholipase A2 | PLA2G10 | O15496 |
| GABA-A receptor; alpha-1/beta-2/gamma-2 | GABRA1 GABRB2 GABRG2 | P14867 P47870 P18507 |
| Estrogen receptor beta | ESR2 | Q92731 |
| Matrix metalloproteinase 9 | MMP9 | P14780 |
| Plasminogen activator inhibitor-1 | SERPINE1 | P05121 |
| Peroxisome proliferator-activated receptor alpha | PPARA | Q07869 |
| Steryl-sulfatase | STS | P08842 |
| NADH-ubiquinone oxidoreductase chain 4 | MT-ND4 | P03905 |
| Glutamate NMDA receptor; GRIN1/GRIN2B | GRIN1 GRIN2B | Q05586 Q13224 |
| Glutamate NMDA receptor; GRIN1/GRIN2A | GRIN2A GRIN1 | Q12879 Q05586 |
| Ornithine decarboxylase | ODC1 | P11926 |
| Free fatty acid receptor 1 | FFAR1 | O14842 |
| Cathepsin (B and K) | CTSB | P07858 |
| Glutamate [NMDA] receptor PROTEIN | GRIN1 | Q05586 |
| Arachidonate 12-lipoxygenase | ALOX12 | P18054 |
| Cathepsin L | CTSL | P07711 |
| Metabotropic glutamate receptor 2 (by homology) | GRM2 | Q14416 |
| Arachidonate 15-lipoxygenase | ALOX15 | P16050 |
| Eukaryotic initiation factor 4A-I | EIF4A1 | P60842 |
| Leukotriene B4 receptor 1 | LTB4R | Q15722 |
| Voltage-gated potassium channel subunit Kv1.3 | KCNA3 | P22001 |
| Aldo-keto-reductase family 1 member C3 | AKR1C3 | P42330 |
| Serotonin 1a (5-HT1a) receptor | HTR1A | P08908 |
| Serotonin transporter | SLC6A4 | P31645 |
| Serotonin 6 (5-HT6) receptor | HTR6 | P50406 |
| Cytochrome P450 2C19 | CYP2C19 | P33261 |
| Purine nucleoside phosphorylase | PNP | P00491 |
| Bile acid receptor FXR | NR1H4 | Q96RI1 |
| Cathepsin K | CTSK | P43235 |
| Sodium/glucose cotransporter 2 | SLC5A2 | P31639 |
| Monoamine oxidase A | MAOA | P21397 |
| Hypoxanthine-guanine phosphoribosyltransferase | HPRT1 | P00492 |
| NAD-dependent deacetylase sirtuin 2 | SIRT2 | Q8IXJ6 |
| Hepatocyte nuclear factor 4-alpha | HNF4A | P41235 |
| Prostanoid EP4 receptor | PTGER4 | P35408 |
| Prostanoid EP2 receptor | PTGER2 | P43116 |
